# Sclerosing mesenteritis affecting the small and the large intestine in a male patient with non-Hodgkin lymphoma: a case presentation and review of the literature

**DOI:** 10.1186/1752-1947-2-388

**Published:** 2008-12-17

**Authors:** Savvas Hiridis, Renos Hadgigeorgiou, Dimitrios Karakitsos, Andreas Karabinis

**Affiliations:** 1 2ndSurgical Department, General Hospital of Athens, 154 Mesogeion Avenue, Athens 11527, Greece; 2 Intensive Care Unit, General Hospital of Athens, 154 Mesogeion Avenue, Athens 11527, Greece

## Abstract

**Introduction:**

Sclerosing mesenteritis is a rare disease resembling a mesenteric tumour. We present here a case of sclerosing mesenteritis that affected both the large and the small intestine of the patient. Therapeutic and diagnostic issues are discussed.

**Case presentation:**

A 62-year-old man with a history of non-Hodgkin lymphoma presented with fatigue, a palpable tender abdominal mass and clinical signs of progressing intestinal obstruction. The preoperative evaluation failed to prove recurrence of the lymphoma or any other definite diagnosis. A laparotomy was performed through a midline incision. The mesentery resembled a tumour-like thickened and fibrotic mass. Abundant, rigid intestinal loop adhesions were observed. Diffuse fibrotic infiltration of the ileum and of the sigmoid colon, which obviously affected the intestinal vascular supply, were identified. A right colectomy and partial sigmoidectomy were performed. Pathological evaluation revealed extensive myofibroblastic reaction of the mesentery with accompanying loci of fat necrosis and areas of inflammation. A diffuse fibrotic infiltration that focally showed a ground-glass appearance was observed. The post-operative course was complicated by respiratory insufficiency and infections and the patient died 2 months after the operation.

**Conclusion:**

Sclerosing mesenteritis that affects both the small and the large intestine is extremely rare. The disease is characterized by myofibroblastic reaction, fat necrosis and diffuse fibrosis of the mesentery. Pathological confirmation may be required for definite diagnosis. If the disease is characterized by severe and diffuse fibrosis, then the application of surgical therapy may be problematic.

## Introduction

Sclerosing mesenteritis (SM) is a rare inflammatory mesenteric disease characterized by chronic fat degeneration and fibrosis; a disease that has become of clinical interest during recent years [1,2]. Some authors described this idiopathic disorder as mesenteric panniculitis (MP), mesenteric lipodystrophy and/or retractile mesenteritis [3-6]. However, these terms may represent histological variants of one single clinical entity [7]. The medical therapy of SM is mostly empiric, and the application of radical surgical therapy remains controversial [2,8]. We present a rare case of SM that affected both the large and the small intestine in a patient with non-Hodgkin lymphoma. Diagnostic and therapeutic issues with emphasis upon the surgical treatment are further discussed.

## Case Presentation

A 62-year-old Caucasian man of Greek origin was admitted to our hospital with a 1-month history of progressive fatigue, intermittent abdominal pain, weight loss, nausea and vomiting. He was diagnosed with a moderately malignant non-Hodgkin B-cell lymphoma 7 years previously. The patient had undergone chemotherapy for a 5-year period followed by a complete remission of the disease. His regular follow-up showed no evidence of recurrence.

**Figure 1:**
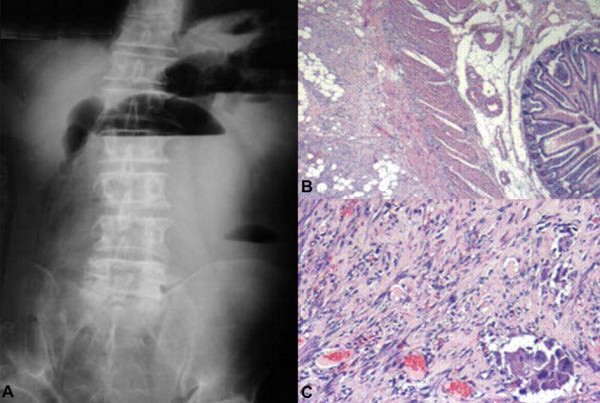
A: Abdominal X-ray of the patient showing dilated intestinal loops and air-fluid levels upon admission. B: Histological section demonstrating zones of myofibroblastic reaction and inflammation, H&E × 63/. C: Histological section depicting loci of fat necrosis and fibrous tissue infiltration that focally bears a ground-glass appearance, H&E × 250/.

Physical examination revealed a palpable tender mass in the left abdomen and scarce abdominal sounds. The rectal examination was negative for blood and laboratory findings were normal. The abdominal X-ray revealed dilated intestinal loops and air-fluid levels (Figure 1A). Computed tomography (CT) showed distended loops of the small intestine. Colonoscopy revealed an erythematous and oedematous appearance of the mucosa but no evidence of inflammatory bowel disease. All pre-operative investigations were inconclusive and the patient's clinical state gradually deteriorated; therefore, a laparotomy was performed through a midline incision. The mesentery was thickened, fibrotic and constricted. There was a small amount of serous effusion in the peritoneal cavity. No enlarged lymph nodes were identified. Abundant, rigid intestinal loop adhesions were observed. The symphysiolysis that was performed proved technically difficult. Diffuse fibrotic infiltration of the ileum and of the sigmoid colon, which obviously affected the intestinal vascular supply were identified. Hence, a right colectomy and a partial sigmoidectomy were performed.

Pathological evaluation revealed an extensive myofibroblastic reaction of the mesentery with accompanying loci of fat necrosis and areas of inflammation (Figure 1B). Also, ischaemic damage of the intestinal mucosa was documented, which could be attributed to the strangulation and thrombosis of the vessels from the fibrotic process. Finally, the histology of this fibrotic infiltration focally exhibited a ground-glass appearance (Figure 1C). Immunohistochemistry showed that the areas of increased myofibroblastic activity were positive for vimentin, desmin and b-cathepsin. Antibodies against CD-34, F-VIII, HHF-35, CK5/6, CD-15, p53 and bcl-2 were negative. Ki67 was positive for a small population of nuclei. CD-3 and L25 were negative for malignant lymphoma. In conclusion, the pathological examination confirmed the diagnosis of SM but failed to reveal any evidence of malignancy [8,9].

Post-operatively, the patient exhibited respiratory insufficiency, thus he was admitted to the intensive care unit. His clinical picture gradually deteriorated and he became septic. Despite prompt antibiotic administration, he died 2-months after the operation.

## Discussion

MP represents an idiopathic inflammatory mesenteric disorder. The disease is known in the literature with many synonyms: mesenteric manifestation of Weber-Christian disease, mesenteric lipodystrophy, mesenteric lipogranuloma and retractile mesenteritis [1-8]. An interesting characteristic of MP is the pathological similarity between the affected fatty tissue and that seen in Weber-Christian disease [10]. SM may be the final stage in the natural course of MP; alternatively, MP and SM may represent histological variants of one single clinical entity [7].

There are fewer than 300 cases of SM reported in the literature since the condition was first described in 1924 [11]. The majority of reported cases of SM involved the small intestine; however, involvement of the large intestine may occur but is extremely rare [1,12,13]. Other rare intra-abdominal sites that were reported to be involved were the mesoappendix, the peripancreatic area, the omentum, the retroperitoneum and the pelvis [12].

The pathogenesis of SM remains unclear. Autoimmune disorders were suggested as a possible mechanism, while other studies suggested that infection, trauma, ischaemia, cold, different drugs, vasculitis, vitamin deficiency or prior surgery could be involved in the pathogenesis [1,7,14]. An association of SM with malignancy, namely lymphoma, breast-, lung- and colon cancer, as well as melanoma has been reported, which could further complicate the diagnosis [1,12,14]. In our case, SM was diagnosed in a patient with a history of non-Hodgkin lymphoma; however, no direct association was proved.

The diagnosis of SM is usually made by biopsy at laparotomy. The presence of a single, multiple or diffuse mass-like inflammatory lesion in the mesentery, together with a histological confirmation of fat necrosis and inflammatory reaction or fibrotic infiltration in the mesenteric lesions, strongly suggests the diagnosis of SM [1,3,6,9]. Magnetic resonance imaging may suggest the fibrous nature of the lesion and delineates vascular involvement [15]. Also, magnetic resonance imaging may show the characteristic features of nonhomogeneous masses of > fat- and soft-tissue density. The mass envelops the mesenteric vessels. Over time, collateral vessels may develop. The CT scan may depict the preservation of fat around the mesenteric vessels, a phenomenon that is referred to as the "fat ring" sign. However, CT cannot distinguish sclerosing mesenteritis from a primary or secondary mesenteric tumour [12,15].

SM is treated largely empirically. Corticosteroids, colchicine, azathioprine, thalidomide, cyclophosphamide and anti-inflammatory agents were suggested as possible therapeutic agents; however, no definite results were documented [1,3,4]. Radical surgery still remains a controversial therapeutic option [1,3,6,8,13]. In a previous report, the authors were in favour of surgical therapy in the earlier stages of the disease [13]. However, with our patient presented here pursuing surgical intervention proved problematic. This was mainly due to the diffuse fibrotic involvement of both the large and the small intestine. This compromized both the vascular supply and the peristaltic mobility of the intestine. Technical issues such as the extensive symphysiolysis, the excision of a large part of the non-peristaltic small intestine, and the difficult mobilization-excision of the affected colon proved that surgical intervention would offer only very limited advantages in the late stages of the disorder.

## Conclusion

SM that affects both the small and the large intestine is extremely rare. The disease might resemble a mesenteric tumour based on clinical, radiological and gross characteristics. Histological confirmation is almost always required. Medical therapy is empiric and surgical therapy remains controversial. If the disease is characterized by severe and diffuse fibrotic infiltration, then the surgical results may be obscure.

## Abbreviations

MP: Mesenteric panniculitis; SM: Sclerosing mesenteritis; CT: Computed tomography

## Consent

Written informed consent was obtained from the next of kin of the patient for publication of this case report and accompanying images. A copy of the written consent is available for review by the Editor-in-Chief of this journal.

## Competing interests

The authors declare that they have no competing interests.

## Authors' contributions

SH and RH both participated in the surgical interventions, contributed to the review of the histological section of the case and drafted the manuscript. DK participated in the medical interventions, took the photographs, undertook the literature review and helped draft the final version of the manuscript. AK participated in all medical interventions and helped draft the final version of this manuscript.

All authors read and approved the final manuscript.
